# A MEG Study of Visual Repetition Priming in Schizophrenia: Evidence for Impaired High-Frequency Oscillations and Event-Related Fields in Thalamo-Occipital Cortices

**DOI:** 10.3389/fpsyt.2020.561973

**Published:** 2020-11-23

**Authors:** Andreas Sauer, Tineke Grent-'t-Jong, Michael Wibral, Michael Grube, Wolf Singer, Peter J. Uhlhaas

**Affiliations:** ^1^Department of Neurophysiology, Max Planck Institute for Brain Research, Frankfurt am Main, Germany; ^2^Singer Lab, Ernst Strüngmann Institute (ESI) for Neuroscience in Cooperation With Max Planck Society, Frankfurt am Main, Germany; ^3^Institute of Neuroscience and Psychology, University of Glasgow, Scotland, United Kingdom; ^4^Department of Child and Adolescent Psychiatry, Charité-Universitätsmedizin Berlin, Berlin, Germany; ^5^Campus Institute for Dynamics of Biological Networks, Georg-August University, Göttingen, Germany; ^6^Department of Psychiatry and Psychotherapy—Psychosomatics, Municipal Clinic, Frankfurt am Main, Germany; ^7^Frankfurt Institute for Advanced Studies (FIAS), Frankfurt am Main, Germany

**Keywords:** neural oscillations, repetition suppression (RS), visual priming, magentoencephalography (MEG), schizophrenia

## Abstract

**Background:** Cognitive dysfunctions represent a core feature of schizophrenia and a predictor for clinical outcomes. One possible mechanism for cognitive impairments could involve an impairment in the experience-dependent modifications of cortical networks.

**Methods:** To address this issue, we employed magnetoencephalography (MEG) during a visual priming paradigm in a sample of chronic patients with schizophrenia (*n* = 14), and in a group of healthy controls (*n* = 14). We obtained MEG-recordings during the presentation of visual stimuli that were presented three times either consecutively or with intervening stimuli. MEG-data were analyzed for event-related fields as well as spectral power in the 1–200 Hz range to examine repetition suppression and repetition enhancement. We defined regions of interest in occipital and thalamic regions and obtained virtual-channel data.

**Results:** Behavioral priming did not differ between groups. However, patients with schizophrenia showed prominently reduced oscillatory response to novel stimuli in the gamma-frequency band as well as significantly reduced repetition suppression of gamma-band activity and reduced repetition enhancement of beta-band power in occipital cortex to both consecutive repetitions as well as repetitions with intervening stimuli. Moreover, schizophrenia patients were characterized by a significant deficit in suppression of the C1m component in occipital cortex and thalamus as well as of the late positive component (LPC) in occipital cortex.

**Conclusions:** These data provide novel evidence for impaired repetition suppression in cortical and subcortical circuits in schizophrenia. Although behavioral priming was preserved, patients with schizophrenia showed deficits in repetition suppression as well as repetition enhancement in thalamic and occipital regions, suggesting that experience-dependent modification of neural circuits is impaired in the disorder.

## Introduction

Schizophrenia is a severe mental disorder characterized by psychotic experiences and disorganized and negative symptoms. In addition, the disorder involves profound deficits in a range of cognitive processes (1). Importantly, cognitive deficits are not modified by current pharmacological treatments and are related to the poor functional outcomes in the majority of schizophrenia patients (2, 3). While cognitive impairments have so far been conceptualized primarily in terms of impairments in higher cognitive functions, such as attention, memory and executive processes (4), there is evidence that dysfunctional sensory processes are also involved (5, 6). Accordingly, the identification of neural mechanisms underlying cognitive impairments in schizophrenia remains one of the major challenges that is important for the development of novel treatments.

Impaired learning mechanisms may contribute to cognitive deficits in schizophrenia as there is evidence of dysfunctional synaptic plasticity (7, 8). Synaptic plasticity refers to synaptic changes in neuronal connections, such as long-term potentiation and depression (LTP/LTD), and is thought to be the primary mechanism for learning and memory (9–12). N-methyl-D-aspartate receptors (NMDA-Rs) play an important role during the induction of LTP/LTD (13). Moreover, there is consistent evidence for an involvement of NMDA-R hypofunctioning in the pathophysiology of schizophrenia (14, 15).

Patients with schizophrenia consistently show deficits in explicit learning and memory (16), while performance on implicit processing tasks appears relatively intact. Thus, studies using motor learning or grammar learning tasks found normal learning (17–19), while probabilistic learning was impaired (18, 19).

One form of implicit memory is repetition priming. Repetition priming refers to improvements in behavioral responses, such as accuracy or reaction time, when stimuli are repeatedly presented (20, 21). Stimulus repetition is associated with changes in single cortical neuron responses (22) as well as in functional magnetic resonance imaging (fMRI)- (23, 24) and in Electro-/Magnetoencephalography data (25, 26). Although stimulus repetition typically leads to repetition suppression, the converse phenomenon, repetition enhancement, has been also been observed in response to unfamiliar stimuli (23) or with low stimulus visibility (27). More recently, it has been shown that repetition suppression and enhancement can occur within one cortical region (28) and that stimulus repetition can induce both enhancement and suppression of neural responses, depending on the number of stimulus presentations (29).

Repetition suppression has been related to the “sharpening” of neural networks (30, 31) which involves a reduction of the number of neurons over successive presentations. More recent studies have shown that top-down processes contribute toward repetition suppression (32, 33). From this perspective, the reduction of neural activity is the result of a comparison between bottom-up (sensory evidence) and top-down activity (predictions), thus leading to a reduction of the prediction error (34, 35).

There is evidence that schizophrenia is associated with impaired repetition suppression. Deficits have been observed for early evoked auditory responses, such as the P50 component (36–40), as well as during pre-pulse inhibition (41–43), in the M170 component in visual cortex (44) and in fMRI-data (45, 46). However, there is also evidence for intact repetition suppression of ERPs (47–49) as well as in fMRI-data (50, 51).

To further investigate repetition priming and the associated neural signatures in schizophrenia, we employed a visual priming paradigm in combination with MEG. Specifically, we presented three visual objects consecutively to examine both repetition suppression and enhancement in MEG-data. In a second experimental condition, stimulus repetitions were interleaved with intervening stimuli to exclude habituation as an alternative explanation for repetition suppression effects.

We focused on the modification of neural oscillations, as beta-/gamma- (52, 53) but also theta-band oscillations (54) provide a temporal structure that allows for precise alignment of the temporal relations between pre- and postsynaptic activation, this relation being crucial in determining the occurrence and the polarity of activity dependent synaptic gain changes (LTP/LTD). More specifically, experience-dependent changes in neural networks appear to depend on the power of stimulus-induced gamma-band oscillations, suggesting a critical role of synchronized gamma-activity for synaptic plasticity (53).

Importantly, there is consistent evidence for aberrant neural oscillations and their synchronization in patients with schizophrenia (55) that could underlie impairments in sensory processing (56–59) as well as higher cognitive functions (60). These data are furthermore consistent with evidence for impaired GABAergic as well as impaired glutamatergic neurotransmission in the disorder (61–64).

Given the important role of neural oscillations in synaptic plasticity (53) as well as the evidence for impaired learning in schizophrenia, we hypothesized that schizophrenia patients would be characterized by impaired behavioral priming as well as reduced repetition suppression in visual cortex as reflected by aberrant gamma-band activity and impaired event-related fields (ERFs) in response to repeated presentation of visual stimuli. Moreover, we also expected close correlations between behavioral impairments, cognitive deficits, and dysfunctional oscillatory activity.

## Methods

### Participants

Fourteen healthy control participants (mean ± SD age, 25.71 ± 4.64 years; 11 females) were recruited from the local community and screened for psychopathology with the German version of the Structured Clinical Interview for DSM-IV-R (SCID) (65). Fourteen patients with schizophrenia (mean ± SD age, 33.79 ± 11 years; three females) were recruited from the out-patient unit of the Psychiatry Department of the Clinic in Frankfurt-Hoechst. All patients fulfilled DSM-IV criteria for schizophrenia as verified by means of a SCID-interview prior to study inclusion. Average disease duration was 10.8 ± 7.1 years. All patients were medicated with atypical neuroleptics. Age and sex differed significantly between both groups (age: t_26_ = −2.68, *p* = 0.012; sex: *X*^2^(1) = 8.31, *p* < 0.01). Current psychopathological symptoms were assessed using the Positive and Negative Syndrome Scale (PANSS) for schizophrenia (66). Symptoms were grouped into five factors – “Negative,” “Positive,” “Excitement,” “Cognitive,” and “Depression” – according to the model of Lindenmayer et al. (67) (Table 1). In addition, patients were also rated on the item “Inappropriate Affect,” which allowed for a score on the factor “Disorganization" (68). Cognitive functions were assessed with the German version of the Brief Assessment of Cognition in Schizophrenia [BACS; (69)] (Table 1). BACS data were standardized (z-transformed) to a normative database, correcting for age and gender (70).

**Table 1:** Demographic, neuropsychological and psychopathological data.

	**Controls (*****n*** **=** **14)**		**SCZ-patients (*****n*** **=** **14)**		**Statistics**
	**Mean**	**SD**	**Mean**	**SD**	
Gender (f/m)	11/3		3/11		*X^2^*(1) = 8.31, *P < * 0.01
Age (years)	25.71	4.64	33.79	11.00	*t*_(26)_ = 2.68, *P < * 0.05
Education (years)	14.86	2.03	14.10	3.31	n.s.
Duration of Illness (years)	—	—	10.80	7.10	—
Handedness, left	0		0		
Behavioral priming					
%-change RT NOLAG	−21.42		−23.11		n.s.
%-change RT LAG	−8.72		−9.20		n.s.
BACS (total)	323.43	21.14	248.21	29.47	*t*_(26)_ = 8.05, *P < * 0.001
Memory	56.57	6.62	40.14	7.96	*t*_(26)_ = 4.37, *P < * 0.001
Digit span	21.50	3.52	16.21	2.58	*t*_(26)_ = 5.15, *P < * 0.001
Token motor	97.86	3.16	81.14	17.71	*t*_(26)_ = 3.12, *P < * 0.005
Fluency	63.86	14.84	45.43	14.27	*t*_(26)_ = 3.27, *P < * 0.005
Symbols	63.86	7.34	49.36	16.20	*t*_(26)_ = 2.54, *P < * 0.05
Tower of London	19.79	1.81	15.93	2.95	*t*_(26)_ = 3.98, *P < * 0.001
PANSS (total)			43.00	24.37	—
Negative	—	—	14.23	5.20	—
Excitement	—	—	6.86	3.91	—
Cognitive	—	—	8.86	2.40	—
Positive	—	—	9.43	3.56	—
Depression	—	—	10.71	2.63	—
Disorganization	—	—	4.64	2.35	—

Participants were excluded if they reported any neurological disorder or current or past alcohol or substance dependence. All subjects were right-handed as assessed by the Edinburgh Handedness Inventory (71) and had normal or corrected-to-normal visual acuity. All participants gave written informed consent prior to the study. The study was carried out according to the declaration of Helsinki and approved by the ethical committee of the Goethe University Frankfurt.

### Stimuli and Task

Stimuli consisted of 200 colored line drawings, picturing natural or man-made objects (72, 73). Stimuli were presented in the center of a translucent screen at a viewing distance of 51 cm and subtended 6.6 degrees of visual angle. A fixation cross was always present in the center of the screen to reduce eye-movements. An LCD projector located outside the magnetically shielded room of the MEG was used to project the stimuli onto the screen via two front-silvered mirrors. Presentation of experimental stimuli was controlled using Presentation (version 14.2, Neurobehavioral Systems, Inc.).

**Figure 1:**
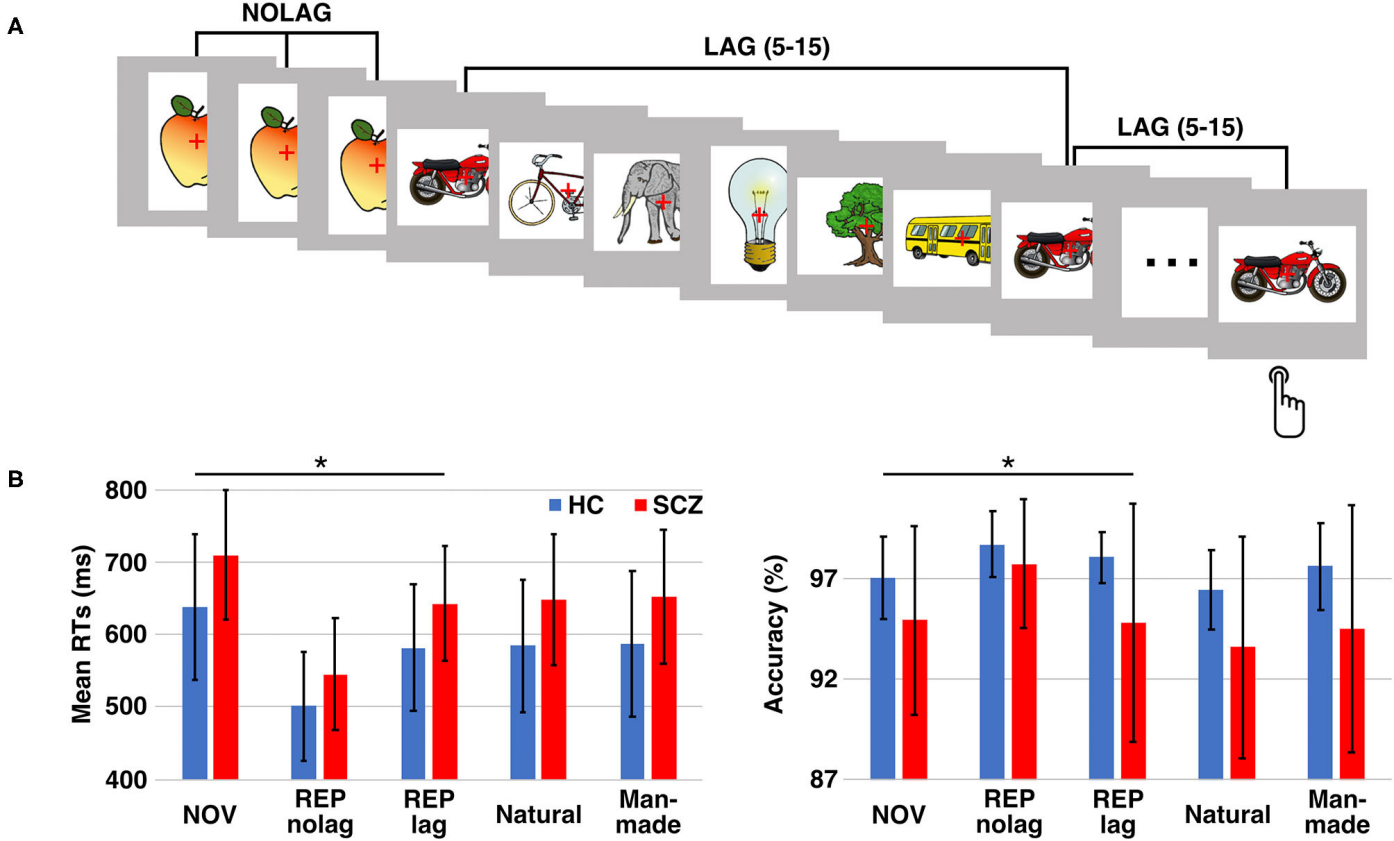
Experimental design and behavioral performance. **(A)** Paradigm: participants had to decide whether the presented object is man-made or natural. Each stimulus was presented three times either in consecutive trials (NOLAG) or with 5–15 different intervening stimuli (LAG). **(B)** Mean response times (RTs; left) and response accuracy (right) ± SD for the different conditions/groups. Asterisk indicates significant group differences (*p* < 0.05, corr.). HC = control participants, SCZ, schizophrenia patients; NOV, Novel; REP, Repeated.

Stimuli were presented for 1000 ms with a randomized inter-stimulus interval of 1000–2000 ms. Each stimulus was presented three times, with either no or different intervening stimuli. The number of intervening stimuli (5–15) was randomized. This resulted in two different repetition conditions: (1) stimulus sequences with three consecutive presentations of the same stimulus without different intervening stimuli (NOLAG) and (2) three presentations of the same stimulus separated by different intervening stimuli (LAG) (Figure 1A). The LAG condition was included to exclude habituation as an alternative explanation for repetition suppression effects and allowed to investigate the modulation of repetition effects by the lag between the repeated presentations of a stimulus (i.e., the “stability” of the repetition effect). Stimulus sequences were shuffled for each participant, resulting in individually randomized stimulus sequences with regards to experimental conditions and stimulus category. Participants were instructed to respond with a button press to indicate whether the presented object was natural or man-made. The assignment of buttons was counterbalanced across participants. Behavioral responses were recorded using a fiber-optic response device (Lumitouch, Photon Control Inc., Burnaby, BC, Canada). Participants were instructed to avoid eye movements and blinking during the presentation of stimuli.

Stimuli were administered in three experimental conditions: (1) novel pictures (i.e., the first presentation of a stimulus; NOV), (2) repeated presentations without lag (NOLAG) and (3) repeated presentations with lag (LAG). The experiment consisted of 600 trials, divided into five blocks of 120 trials each.

### Neuroimaging

MEG data were acquired using a 275-sensor whole-head system (Omega 2005, VSM MedTech Ltd.) with a sampling rate of 600 Hz in a synthetic third order axial gradiometer configuration. Before and after each block, head position relative to the gradiometer array was measured. Recordings with head movements exceeding 5 mm were discarded. A high-resolution anatomical MRI scan was acquired for each participant on a 3 T Siemens Trio scanner, using a 3D-MPRAGE sequence (160 slices, voxel size: 1 × 1 × 1 mm; field of view: 256 mm, repetition time: 2300 ms, echo time: 3.93 ms), with markers placed at the same locations as the sensors used for recording head position in the MEG. These markers were used for subsequent co-registration of the MEG data to the anatomical T1 image.

### MEG Data Analysis

MEG data were analyzed with MATLAB using the open source Fieldtrip toolbox (74). Trials were defined from the continuously recorded MEG signal from −1000 to 1500 ms with respect to the onset of the stimulus. Pre-processing included lowpass filtering of the MEG data (Butterworth filter fourth order) with a lowpass frequency of 200 Hz as well as detrending. Power line fluctuations (50 Hz and harmonics) and a 60 Hz beamer noise signal were removed using a band-stop filter. Independent component analysis (ICA) was used to remove artifacts due to cardiac activity, eye movements and eye blinks. Trials containing muscle artifacts or sensor (SQUID) jumps were discarded using semi-automatic artifact rejection routines. Data epochs for NOV trials were pooled together from the NOLAG and LAG conditions, and only trials with correct responses were considered for further analyses.

All analyses were conducted on “virtual channel” reconstructed MEG data. Linearly constrained minimum variance (LCMV) beamformer spatial filters (75) were used to first reconstruct the MEG data from MNI source locations corresponding to centroids of 80 of 116 available AAL atlas regions (76).

Time-frequency power representations (TFRs) were computed on the LCMV reconstructed time-series, using a sliding window Fast Fourier Transform (FFT) approach with a fixed window of 200 ms and a step size of 10 ms across the length of the epochs. Power of all frequencies between 1 and 200 Hz was estimated based on data padded up to 4 s, using a frequency resolution of 1 Hz, and multiplying the data with a Hanning taper before power estimation.

To analyze ERFs, trials were low-pass filtered at 20 Hz (Butterworth filter fourth order) and baseline corrected using the first 200 ms of each epoch. Trials were averaged per condition and per participant. Based on previous studies (25, 77–79) ERFs were analyzed for early components C1m (30–80 ms) and M100 (80–120 ms) as well as the P300m (200–400 ms) and the late positive component (LPC, 400–600 ms).

Fourteen regions were included in the further analyses: 12 covering striate and extra-striate visual-cortical areas based on previous studies (25, 78) and two (bilateral) thalamic regions (see **Figures 4A,B**).

### Statistical Analyses

Behavioral data were analyzed by means of repeated measures ANOVA using JASP (version 0.13.1; University of Amsterdam, NL). A probability level of *p* < 0.05 was considered as statistically significant. Statistical analyses of MEG data for factors GROUP (Controls vs. Patients), NOVELTY (Novel vs. Repetition) and PRESENTATION (NOLAG vs. LAG) were assessed using non-parametric Monte-Carlo permutation independent F-tests as implemented in Fieldtrip with 1999 permutations and cluster-based correction for multiple comparisons (*p* < 0.05, one sided). To examine differences between experimental conditions within and between groups, non-parametric Monte-Carlo permutation *t*-test statistics with 1999 permutations and cluster-based correction for multiple comparisons (*p* < 0.05, two sided) as implemented in Fieldtrip were performed. Cluster-based statistics were computed across the 12 occipital cortex regions of interest (ROIs) and for the two thalamic ROIs, separately. Effects of picture repetition on modulation of neural activity were statistically evaluated within each group in relation to the response to novel stimuli, with contrasting novel stimuli (NOV) against repeated stimuli for each repetition condition (NOLAG or LAG). For group effects, the difference between NOV and REP for each repetition condition (NOLAG or LAG) was calculated within each group and then contrasted against each other. As sex and age were significantly different between groups, the influence of these variables on group differences of repetition effects was analyzed with correlational analyses (see Supplementary Figure 2).

## Results

### Neuropsychological Data

Schizophrenia patients were significantly impaired in the BACS-total score as well as in each of the subtests (t = 7.76, *p* < 0.001; mean z-score ± SD, controls: 4.33 ± 2.29, schizophrenia patients: −4.33 ± 3.31; Table 1).

### Behavioral Data

Analysis of reaction times (RTs) revealed a main effect of GROUP [*F*_(1, 27)_ = 13.97, *p* < 0.001], a main effect of NOVELTY [*F*_(1, 27)_ = 41.12, *p* < 0.001], and a main effect of PRESENTATION [*F*_(1, 27)_ = 7.18, *p* < 0.01]. Tukey *post-hoc* analyses showed that schizophrenia patients responded significantly slower than controls (GROUP: t = −3.72, *p* < 0.001, mean difference: −71.20 ms, 95%-CI [−132.22, −10.17]; mean RT ± SD, controls: 638.62 ± 100.44 ms, schizophrenia patients: 709.82 ± 89.07 ms). Moreover, RTs were faster for repeated presentations (NOVELTY: t = 6.41, *p* < 0.001, mean difference: 106.47 ms, 95%-CI [73.55, 139.40]; mean RT ± SD, NOV: 674.22 ± 99.95 ms, REP: 567.75 ± 93.83 ms), and that participants responded faster for presentations without different intervening stimuli (PRESENTATION: t = −3.72, *p* < 0.001, mean difference: −44.48 ms, 95%-CI [−77.41, −11.56]; mean RT ± SD, NOLAG: 598.74 ± 117.23 ms, LAG: 643.23 ± 98.16 ms) (Figure 1B). There was no GROUP × NOVELTY interaction, indicating a comparable behavioral priming effect to repeated presentations in the two groups [*F*_(1, 27)_ = 0.33, *p* = 0.569] (Table 1). A significant NOVELTY × PRESENTATION interaction indicated that RTs were significantly different between novel and repeated presentation depending on the repetition condition [*F*_(1, 27)_ = 7.18, *p* < 0.01].

Analyses of accuracy showed a main effect of GROUP [*F*_(1, 27)_ = 9.86, *p* < 0.005]. Tukey *post-hoc* analysis showed that accuracy was higher in controls compared to schizophrenia patients (t = 3.14, *p* = 0.002; mean accuracy ± SD, controls: 98.37 ± 0.98%, patients: 96.69 ± 3.30%; Figure 1B).

### MEG Data

#### Responses to Novel Stimuli

**Figure 2:**
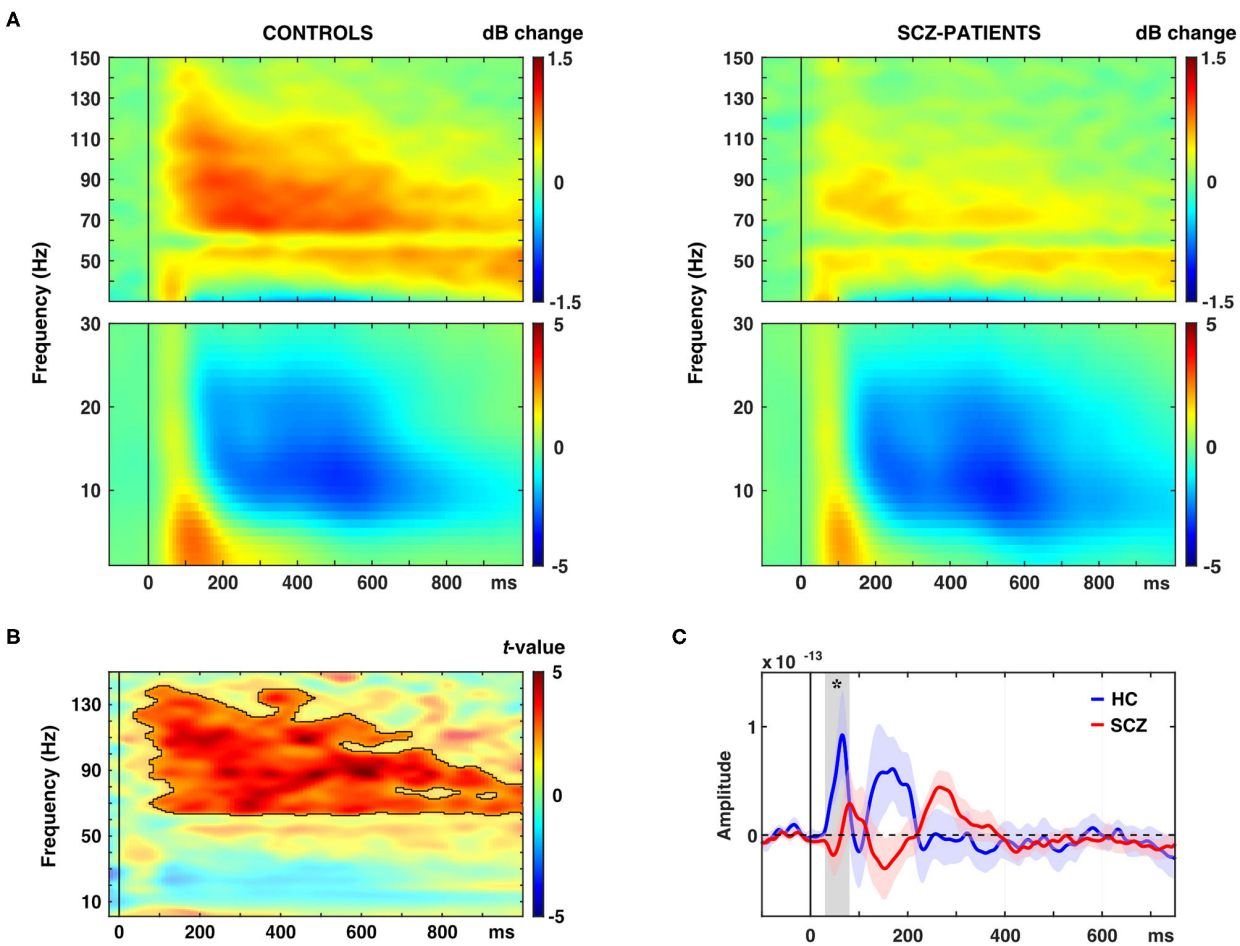
Effects of novel pictures on spectral power and event-related fields (ERFs). **(A)** Time-frequency responses (TFR) of AAL atlas reconstructed virtual channel data. TFR show grand average (*N* = 14 per group) data from across 12 regions in the occipital cortex as displayed in Figure 4A (averaged dB power changes from baseline) for novel picture presentations in control participants (left) and schizophrenia patients (right). **(B)** TFR plot with statistical result (non-parametric, Monte-Carlo permutation independent *t*-test, cluster corrected, *p* < 0.05) of group difference with significant cluster outlined. **(C)** ERF time-course of grand average (*N* = 14 per group) data in the occipital cortex for novel picture presentations in control participants (HC) and schizophrenia patients (SCZ). Asterisk indicates a significant difference between groups (cluster corrected, *p* < 0.05).

The schizophrenia group was characterized by a significantly reduced oscillatory response in occipital ROIs in the gamma-band to novel stimuli (63–141 Hz, 0.04–1.0 s, t_sum_ = 14,470, *p* = 0.001, 95%-CI [−0.0004, 0.0024]; Figures 2A,B, Table 2). This effect was observed in bilateral cuneus, calcarine sulci as well as bilateral superior, middle and inferior occipital gyri. Moreover, schizophrenia patients showed a significantly reduced C1m component in occipital ROIs (t_sum_ = 25.45, *p* = 0.036; Figure 2C, Table 2).

**Table 2:** Summary of significant within- and between-group effects in MEG data.

**Effect**	**ROI**	**Frequency range**	**ERF latency**	***P***
**CONTROLS**				
NOV vs. REPnolag	Occipital ROI	42–123 Hz2–35 Hz		0.0010.008
	Occipital ROI		30–80 ms	0.029
			400–600 ms	0.020
	Thalamus		30–80 ms	0.053
NOV vs. REPlag	Occipital ROI	42–129 Hz		0.001
		1–36 Hz		0.003
	Occipital ROI		30–80 ms	0.031
**SCZ-PATIENTS**				
NOV vs. REPnolag	Occipital ROI	66–88 Hz		0.016
		1–37 Hz		0.001
NOV vs. REPlag	Occipital ROI	12–40 Hz		0.009
**CON vs. SCZ**				
NOV vs. REPnolag	Occipital ROI	43–98 Hz		0.001
		14–33 Hz		0.039
	Occipital ROI Thalamus		30–80 ms30–80 ms	0.0050.019
NOV vs. REPlag	Occipital ROI	50–98 Hz		0.015
		12–31 Hz		0.024
	Occipital ROI		30–80 ms	0.063
NOV	Occipital ROI	63–141 Hz		0.001
			30–80 ms	0.036

#### Repetition Suppression/Enhancement

##### Spectral power

**Figure 3:**
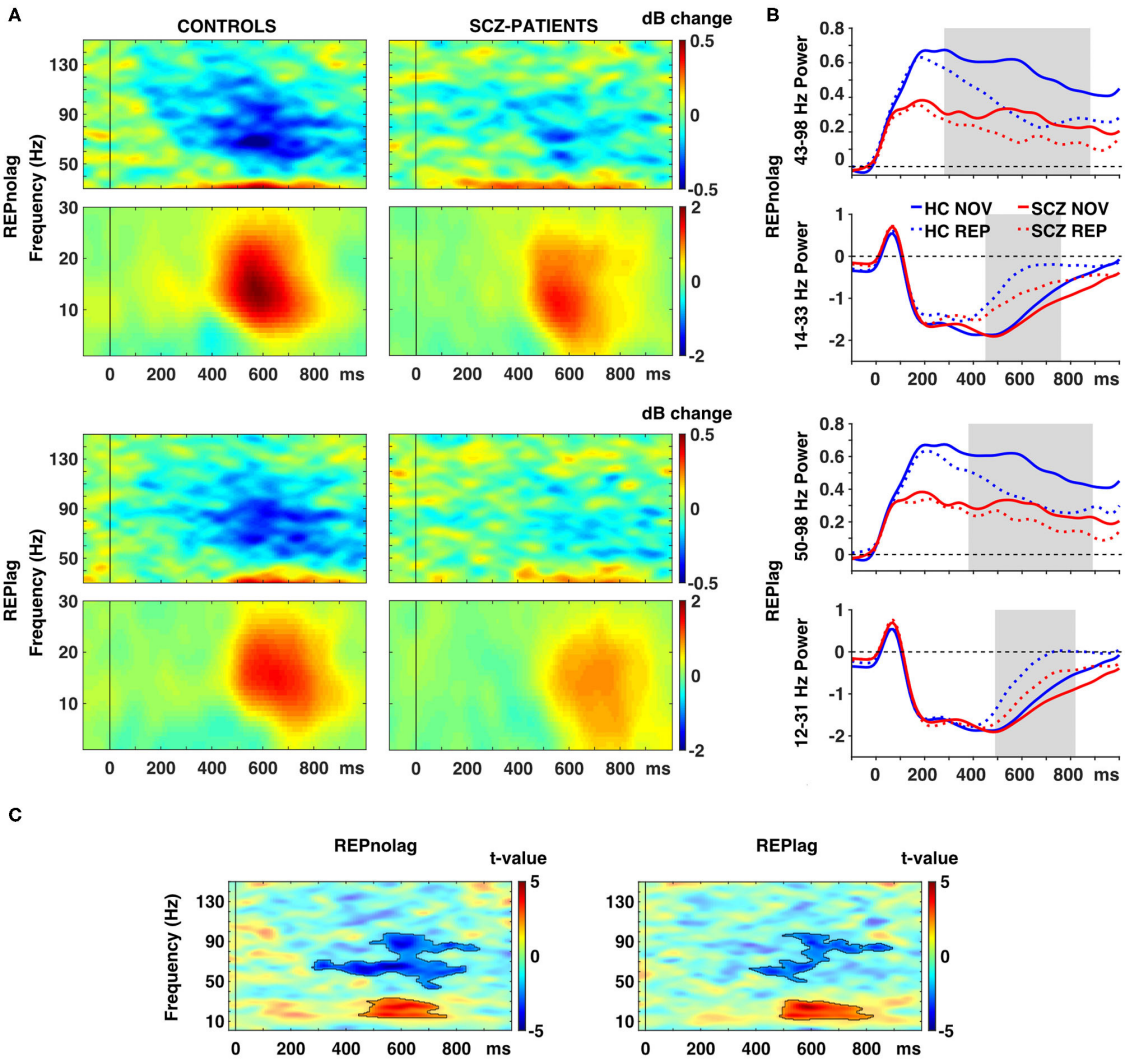
Effects of picture repetition on spectral power. **(A)** Time-frequency responses (TFR) of AAL atlas reconstructed virtual channel data. TFR show grand average (*N* = 14 per group) data from across 12 regions in the occipital cortex as displayed in Figure 4A (averaged dB power changes from baseline) for the difference between novel and repeated pictures (REP minus NOV) for repetitions with no lag (first row) and lag (second row) in control participants (left) and schizophrenia patients (right). **(B)** Line graphs show averaged power for gamma (top) and beta (bottom) frequencies over time per group for novel and repeated picture presentations in the NOLAG (first row) and LAG (second row) condition. **(C)** TFR plots with statistical results (non-parametric, Monte-Carlo permutation independent *t*-test, cluster corrected, *p* < 0.05) of group difference for the effect of repetition (REP minus NOV) with significant clusters outlined. HC, control participants; SCZ, schizophrenia patients; NOV, novel; REP, repeated.

Analyses of factors GROUP, NOVELTY, and PRESENTATION revealed a main effect of GROUP (*p* < 0.001, *F*_sum_ = 110785.70), a main effect of NOVELTY (*p* = 0.012, *F*_sum_ = 20049.06), but no main effect of PRESENTATION (*p* = 0.9, *F*_sum_ = 27.95). *Post-hoc* pairwise *t*-test analyses revealed that stimulus repetitions led to a significant reduction of gamma-band power in occipital cortex ROIs in controls in both NOLAG (42–123 Hz, t_sum_ = −11366, *p* = 0.001; Figures 3A,B) and LAG (42–129 Hz, t_sum_ = −8903.30, *p* = 0.001; Figures 3A,B) conditions (Table 2). In the NOLAG condition, the effect was observed in all occipital cortex ROIs, whereas the effect in the LAG condition was observed only in right cuneus, right inferior occipital gyrus as well as bilateral calcarine sulci and superior and medial occipital gyri. Schizophrenia patients, however, showed a significant reduction of gamma-band power to repeated presentations only in the NOLAG condition and with a smaller bandwidth (66–88 Hz, t_sum_ = −1262.30, *p* = 0.016; Figures 3A,B, Table 2). Furthermore, this effect was spatially limited to the right lingual gyrus.

Importantly, schizophrenia patients showed an impaired repetition suppression of gamma-band power in both NOLAG (43–98 Hz, t_sum_ = −3319.10, *p* = 0.001; Figure 3C) and LAG (50–98 Hz, t_sum_ = −1658.70, *p* = 0.015; Figure 3C) conditions in occipital ROIs compared to controls (Table 2). For both conditions, impaired repetition suppression of gamma-band activity was observed in bilateral cuneus, calcarine sulci as well as bilateral superior occipital gyri.

In frequencies <40 Hz, both groups showed repetition enhancement in occipital cortex ROIs to repeated presentations in both NOLAG (controls: 2–35 Hz, t_sum_ = 4,514, *p* = 0.008; schizophrenia patients: 1–37 Hz, t_sum_ = 2957.70, *p* = 0.001; Figures 3A,B) and LAG (controls: 1–36 Hz, t_sum_ = 4632.2, *p* = 0.003; schizophrenia patients: 12–40 Hz, t_sum_ = 1972.10, *p* = 0.009; Figures 3A,B) conditions (Table 2). For controls, the effect was observed in all occipital cortex ROIs in both NOLAG and LAG conditions. Schizophrenia patients, on the other hand, showed repetition enhancement of beta-band activity only in bilateral cuneus, calcarine sulci, superior and medial occipital gyri and the right lingual gyrus.

Repetition enhancement of beta-band-power was significantly reduced in the schizophrenia group compared to controls in both NOLAG (14–33 Hz, t_sum_ = 1238.70, *p* = 0.039; Figure 3C) and LAG (12–31 Hz, t_sum_ = 1407.20, *p* = 0.024; Figure 3C) conditions in occipital ROIs (Table 2). Group differences for repetition enhancement of beta-band power were found in bilateral calcarine sulci and the right superior occipital gyrus in the NOLAG condition and in the right calcarine sulcus as well as the left middle occipital gyrus in the LAG condition.

##### ERFs

**Figure 4:**
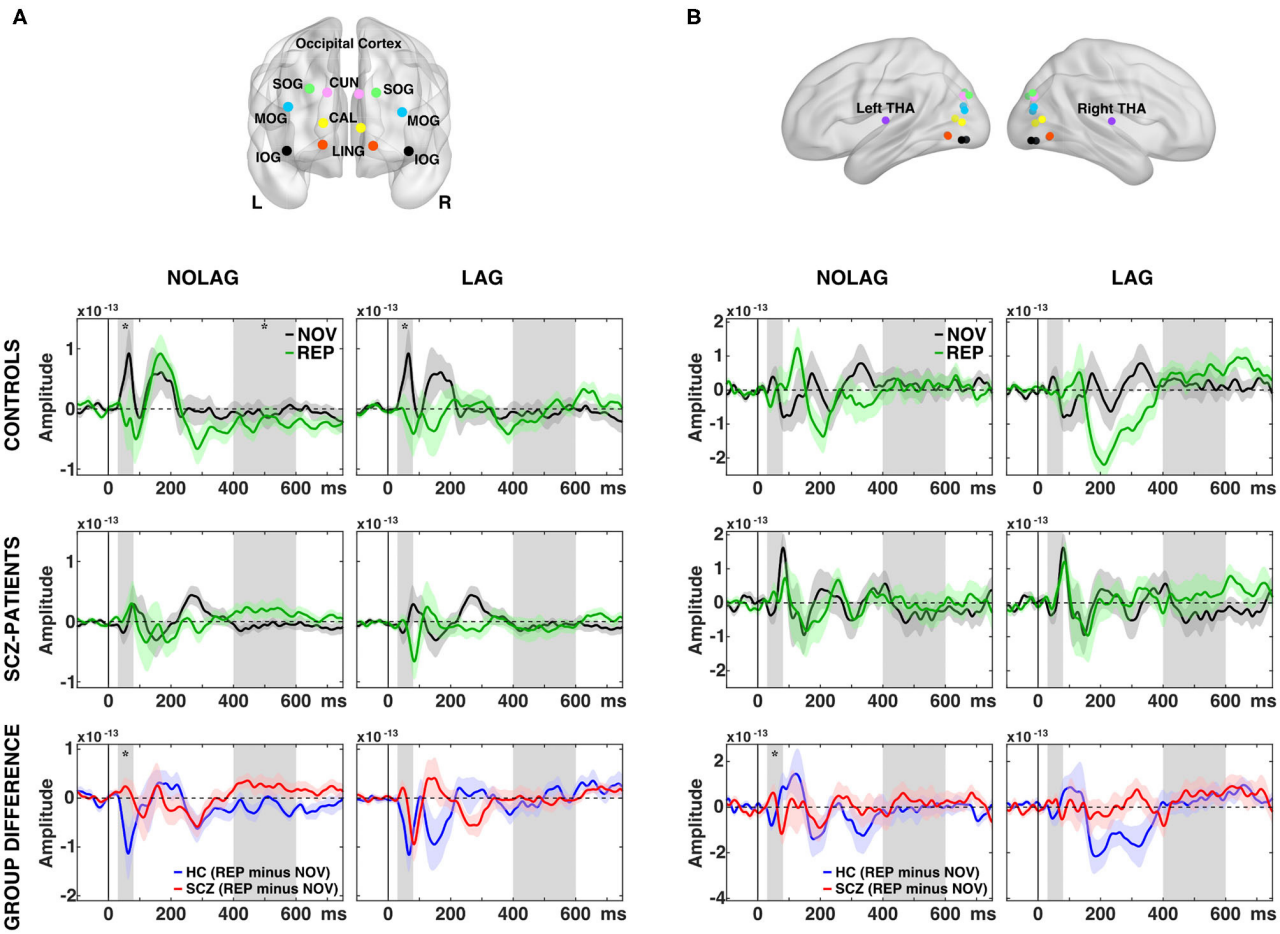
Effects of picture repetition on event-related fields (ERFs). **(A)** Indication of regions of interest (ROI) used in the analysis for the occipital cortex, projected onto a transparent MNI template brain. CAL, calcarine sulcus; CUN, cuneus; LING, lingual gyrus; SOG/MOG/IOG, superior/middle/inferior occipital gyrus. ERF time-courses of grand average (*N* = 14 per group) data from across 12 regions in the occipital cortex for consecutively repetitions (NOLAG, left) and repetitions with intervening different stimuli (LAG, right) for control participants (first row) and schizophrenia patients (second row). Third row shows the group difference for the effect of repetition (REP minus NOV within group). **(B)** As in **(A)** but for the thalamus = THA. HC, controls participants; SCZ, schizophrenia patients; NOV, Novel; REP, Repeated. Gray boxes indicate the time window used for the statistical analyses (C1m = 30–80 ms; LPC = 400–600 ms). Asterisk indicates a significant difference between conditions/groups (cluster corrected, *p* < 0.05).

Analyses of factors GROUP, NOVELTY and PRESENTATION showed no significant main effects. *Post-hoc* pairwise *t-*tests revealed that controls showed an early effect of stimulus repetition with a significant reduction of the C1m (30–80 ms) component in the NOLAG and LAG condition (NOLAG: t_sum_ = 19.83, *p* = 0.029; LAG: t_sum_ = 43.90, *p* = 0.031) in all occipital ROIs (Figure 4A, Table 2). In addition, we observed a statistical trend in the NOLAG condition in the thalamus (t_sum_ = 24.20, *p* = 0.053; Figure 4B, Table 2). Importantly, repetition suppression of the C1m was not present in schizophrenia patients neither in the NOLAG nor the LAG conditions across occipital and thalamic ROIs (Figures 4A,B). There was a significant group difference in C1m repetition suppression in the NOLAG condition in occipital ROIs (t_sum =_ −43.70, *p* = 0.005) and thalamus (t_sum =_ −32.36, *p* = 0.019) but only as a statistical trend in the LAG condition in occipital ROIs (t_sum =_ −20.39, *p* = 0.063) (Figures 4A,B, Table 2). The group effect in the NOLAG condition was mainly driven by the left calcarine sulcus and left superior occipital gyrus as well as the left thalamus, respectively, while the group effect in the LAG condition was observed only in the left calcarine sulcus.

In addition, we observed repetition suppression of the LPC in occipital ROIs in the NOLAG condition in controls (400–600 ms, t_sum_ = 82.39, *p* = 0.02; Figure 4A). In schizophrenia patients, this effect did not reach statistical significance (t_sum_ = −56,19, *p* = 0.09), and the group difference showed only a trend toward statistical significance when averaged over all occipital ROIs (t_sum_ = −61.70, *p* = 0.058). However, there was a significant difference in right calcarine sulcus (tsum = −259.89, *p* = 0.002).

Analyses of the M100 (80–120 ms) and P300m (200–400 ms) revealed no significant differences within or between groups.

### Correlations With Behavior and Psychopathology vs. MEG Data

Pearson's correlations were used on z-normalized data to investigate relationships between repetition suppression/enhancement of the C1m, spectral power in the low (12–33 Hz) and high frequency (43–98 Hz) range and response times, accuracy and PANSS ratings. Bootstrapping (1000 randomizations) was applied to control for spurious findings. We did not find any significant correlations between repetition suppression/enhancement and behavioral parameters in both groups as well as with psychopathology in schizophrenia patients.

## Discussion

The present study investigated repetition suppression in schizophrenia with MEG to examine changes in neural oscillations and ERFs during visual priming. While behavioral priming was intact in schizophrenia patients, we observed a dysregulation of both low- and high-frequency oscillations as well as impaired ERFs, suggesting an impairment in the experience-dependent modification of neural circuits.

Consistent with previous studies that examined implicit learning in schizophrenia (80, 81), we found no difference in behavioral priming between schizophrenia patients and controls. However, differences between groups emerged in MEG-parameters, suggesting a dissociation between repetition suppression/enhancement and behavioral priming. This is also supported by the fact that there were no significant correlations between MEG-data, cognitive and clinical variables. Moreover, previous studies failed to show a significant correlation between behavioral priming and neural suppression in occipital cortex during normal brain functioning (82–85), raising the possibility that behavioral priming and repetition suppression are distinct processes.

Consistent with previous studies that investigated repetition effects on neural oscillations and ERPs in healthy participants using visual paradigms (25, 78, 86), we observed repetition suppression of gamma-band frequencies and ERF components as well as repetition enhancement of beta-band frequencies in normal controls. Importantly, our data highlight an impairment in high-frequency oscillations in patients with schizophrenia during responses to novel stimuli as well as aberrant modulation of beta- and gamma-band activity during repetition suppression and enhancement. Consistent with a large body of EEG/MEG-data that demonstrated reduced power of high-frequency oscillations during sensory and perceptual processing (56, 59, 87, 88), we observed a pronounced (effect size: d = 1.48) and sustained reduction of high-gamma (>60 Hz) power that was extended over a large frequency range and time interval in occipital areas belonging to both dorsal and ventral processing streams.

Furthermore, modulation of spectral power during repetition suppression and enhancement at beta/gamma-band frequencies was also impaired in schizophrenia patients. Because of the pronounced reduction of gamma-band power to novel stimuli in schizophrenia patients, we have specifically examined repetition effects in relation to the response to novel stimuli within each group. Although the reduced gamma-band response to novel stimuli limits the range of repetition suppression in schizophrenia patients, our finding of impaired repetition enhancement of beta-band oscillations without a decreased beta-band response to novel stimuli, indicates a general deficit in repetition-related modulation of spectral power in schizophrenia patients.

Recently, Galuske et al. (53) have shown that changes of neuronal response properties induced by repetitive visual stimulation depend on the magnitude of induced gamma-band oscillations. The authors observed changes in orientation tuning of neurons in visual cortex only when conditioning stimuli induced strong gamma-oscillations, suggesting a critical role of synchronized gamma-oscillations for facilitating experience-dependent plasticity. Accordingly, aberrant repetition suppression in schizophrenia patients might result from a persistent failure to generate gamma-band oscillations that in turn impairs synaptic plasticity.

As perception is dependent upon inferential processes whereby sensory evidence is weighted against prior knowledge (34, 89), it is possible that shallow processing, as indexed by reduced gamma-band responses to the initial stimulus, leads to a weaker formation of the prior in patients with schizophrenia. In the context of predictive coding, repetition suppression reflects the attenuation of the prediction error due to the decreasing mismatch between predictions and sensory input. As the magnitude of the prediction error signal reflects the match between prior and sensory data, weak priors in schizophrenia patients might result in a greater mismatch and thus in reduced repetition suppression. Interestingly, in the schizophrenia group repetition suppression effects were only observed in the NOLAG condition. This could index that interfering stimuli in the LAG condition further diminished the impact of the prior, thus leading to a further reduction of repetition suppression. In agreement with this interpretation is the recent finding (90) that the amplitude of induced gamma oscillations is positively correlated with the goodness of the match between sensory evidence and internal predictions.

Different oscillation frequencies have been related to different components of inferential processing. Top-down prediction signaling has been proposed to be predominantly mediated by alpha-/beta-frequencies and feed-forward prediction error signaling by gamma- and theta-band oscillations (91, 92). As group differences in gamma-band activity emerged in early visual areas, such as cuneus and calcarine sulcus, as well as higher-order visual regions, such as superior occipital gyrus, reduced gamma-band activity in patients with schizophrenia may lead to reduced feed-forward signaling as recently demonstrated by our group (59). According to the predictive coding framework, the predictive model at higher levels of the hierarchy is updated by the ascending prediction error, which has more impact on the prior when it conveys precise information (through precision weighting). Reduced gamma responses in patients with schizophrenia may lead to a deficient updating of priors through reduced precision of the prediction error signal resulting from imprecise sensory data input.

This is in line with our finding of impaired repetition enhancement of beta-band activity in schizophrenia patients. Precision of predictions increases as prediction error is minimized by repetitions, which may underlie repetition enhancement (35). Reduced repetition enhancement in schizophrenia patients could index reduced precision of predictions, which may result from a deficient interplay between top-down predictions and bottom-up prediction error signaling.

Consistent with a previous report (79) we also found that stimulus repetition was associated with an early (30–80 ms) reduction of the C1m component in healthy controls in visual cortex. The C1 component reflects the first visual evoked potential (VEP) component, with an onset latency between 40 and 70 ms and peak latency between 60 and 100 ms, and originates from primary visual cortex in striate cortex within the calcarine fissure (93–97). Importantly, repetition suppression of this early VEP was impaired in schizophrenia patients. This finding is in line with a large body of research on early-stage visual processing deficits in schizophrenia (59, 98).

Furthermore, we found impaired repetition suppression of early VEPs in schizophrenia patients in the thalamus. The thalamus plays a key role in information processing as nearly all sensory information must pass the thalamus before reaching the cerebral cortex, and there is consistent evidence for both anatomical (99) and functional abnormalities (100, 101) in schizophrenia.

In addition, we observed impaired repetition suppression of the LPC in the schizophrenia group in occipital ROIs. The LPC has been associated with recognition memory processes [“old/new effect;” (102)]. Specifically, Matsuoka et al. (103) using repetition priming found that schizophrenia patients showed no effect of immediate stimulus repetition on late ERPs that could index a failure to use information from preceding stimuli.

However, these deficits between groups only reached trend level in the LAG condition, which is consistent with previous studies showing that suppression of ERPs (78, 104) dissolves with lag between repetitions.

## Limitations

There are several limitations associated with this study. Firstly, we only included a relatively small sample of patients with schizophrenia. As a result, the lack of significant correlations between behavioral and neuroimaging measures might be due to insufficient statistical power. In addition, we did not systematically assess the contribution of eye movements toward differences in both behavior and MEG-data. Finally, observations in deep brain structures like the thalamus with MEG can be challenging because of the decay of the magnetic field. However, the large effect size (d = 0.98) of our result support the feasibility of MEG in combination with individual anatomical information to assess thalamic signals (101, 105, 106).

## Summary

The present study provides novel evidence for impaired repetition suppression and repetition enhancement in schizophrenia as reflected by deficits in the experience-dependent modification of beta/gamma-band oscillations as well as ERFs during visual priming. Specifically, schizophrenia patients showed impaired repetition suppression of early and late evoked visual responses as well as gamma-band oscillations. In the context of predictive coding, reduced gamma-band activity may lead to impaired feed-forward signaling which could then lead to reduced repetition suppression and enhancement.

Since deficits in repetition suppression have been found to be present even before the onset of the disorder (107, 108), it will be important to further investigate neural mechanisms of repetition suppression and their impairment in at-risk populations as well as in larger cohorts of schizophrenia patients to examine whether effects of reduced repetition suppression involve aberrant connectivity between cortical areas.

## Data Availability Statement

The raw data supporting the conclusions of this article will be made available by the authors, without undue reservation.

## Ethics Statement

The study was approved by the ethical committee of the Goethe University Frankfurt. The patients/participants provided their written informed consent to participate in this study.

## Author Contributions

AS contributed to design, recordings, analyses, and write-up of the study. TG-'t-J contributed to analyses and write-up of the study. MW, MG, WS, and PU (leading investigator) are senior scientists who supervised the entire study, from design to final submission.

## Conflict of Interest

The authors declare that the research was conducted in the absence of any commercial or financial relationships that could be construed as a potential conflict of interest.
