# Supplementary figures and images for: A MEG Study of Visual Repetition Priming in Schizophrenia: Evidence for Impaired High-Frequency Oscillations and Event-Related Fields in Thalamo-Occipital Cortices
Source: Front Psychiatry. 2020 Nov 23;11:561973. doi: 10.3389/fpsyt.2020.561973 (PMC7719679; doi:10.3389/fpsyt.2020.561973)

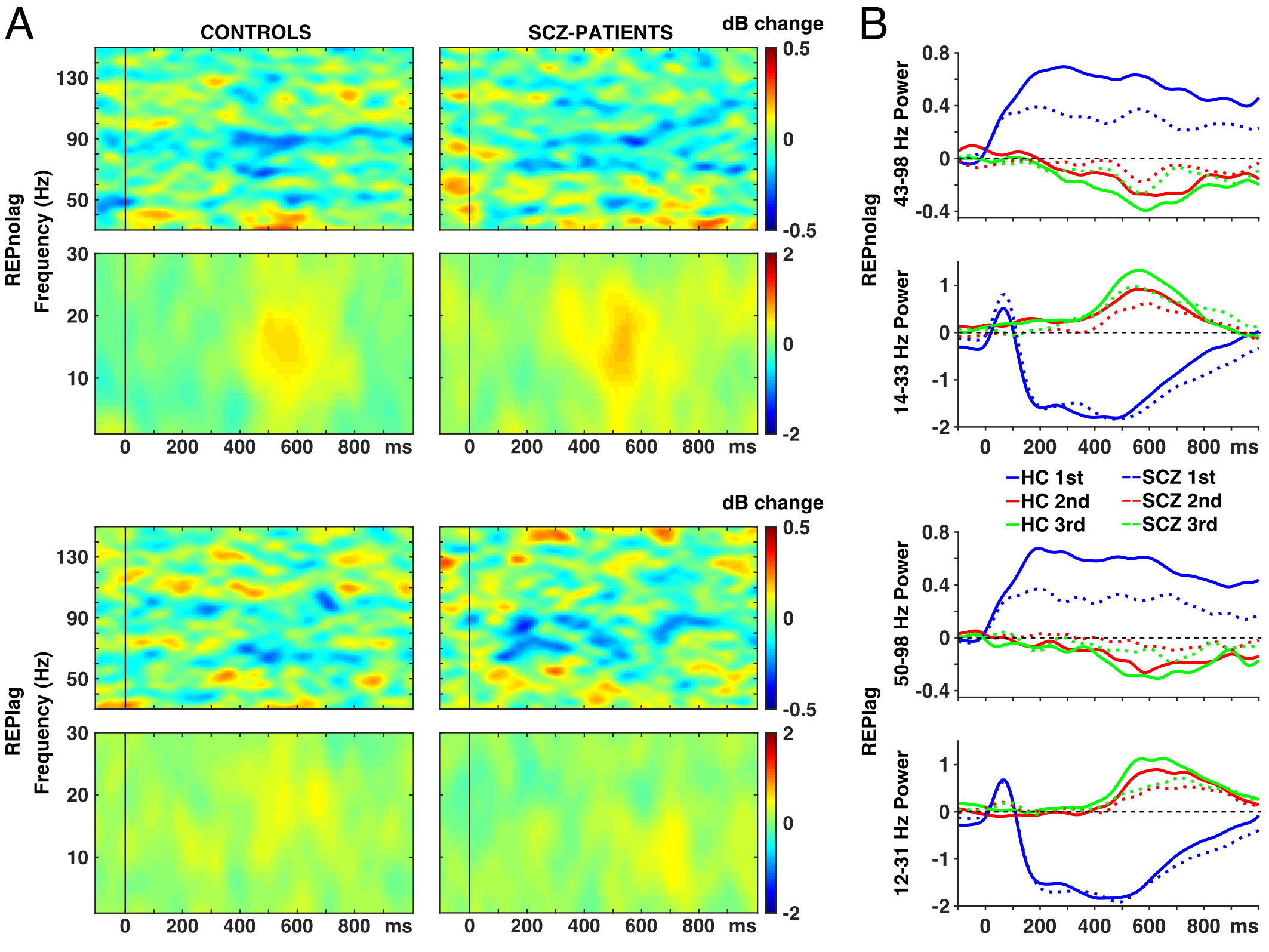

Supplement: Supplementary file 1 [file Image_1.TIFF]

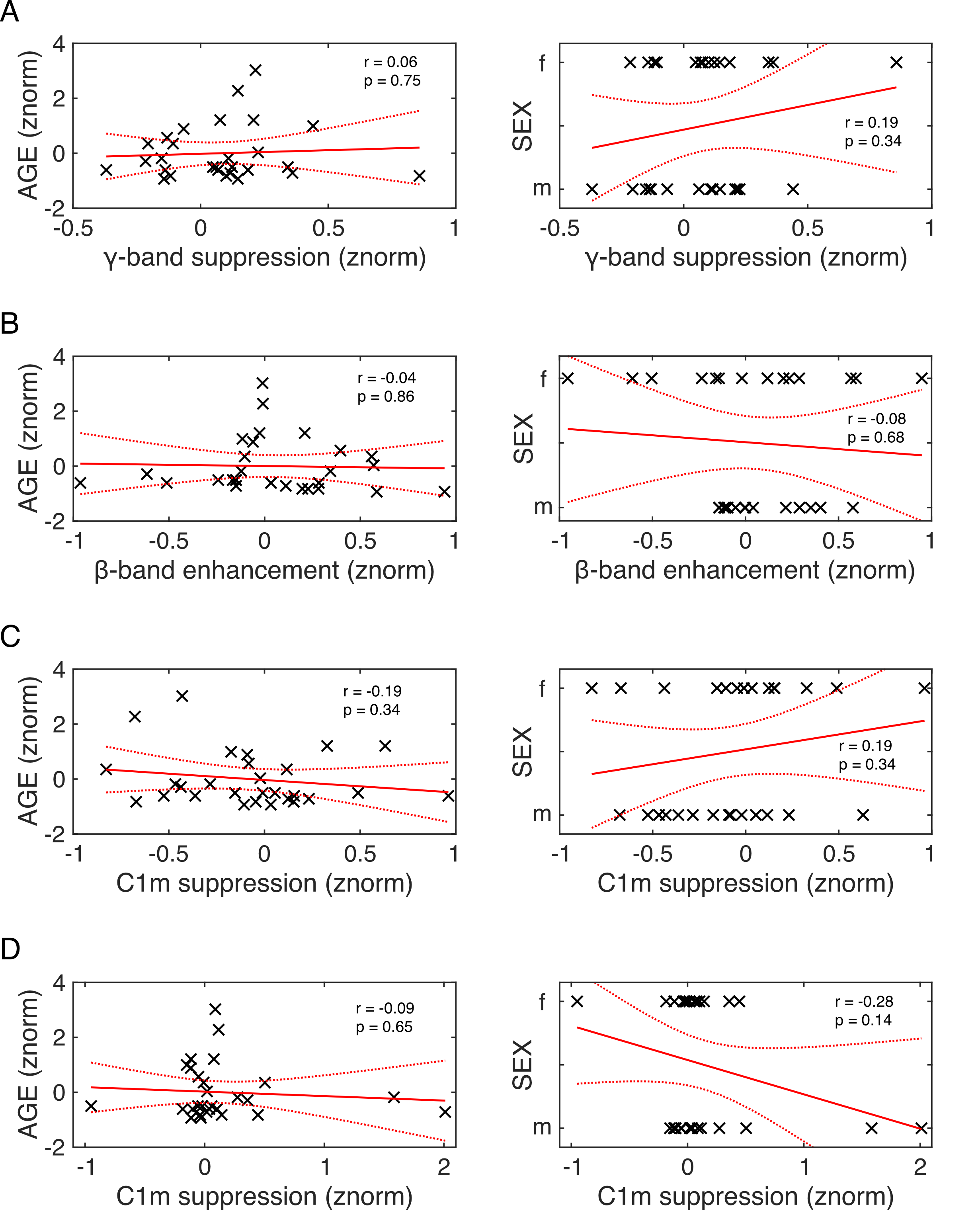

Supplement: Supplementary file 2 [file Image_2.TIFF]
