# Supplementary material for: A MEG Study of Visual Repetition Priming in Schizophrenia: Evidence for Impaired High-Frequency Oscillations and Event-Related Fields in Thalamo-Occipital Cortices
Source: Front Psychiatry. 2020 Nov 23;11:561973. doi: 10.3389/fpsyt.2020.561973 (PMC7719679; doi:10.3389/fpsyt.2020.561973)
Supplement: Supplementary file 3 [file Data_Sheet_1.docx]

Supplementary Figure 1. Effects of picture repetition on spectral power. (A) Time-frequency responses (TFR) of AAL atlas reconstructed virtual channel data. TFR show grand average (N = 14 per group) data from across 12 regions in the occipital cortex as displayed in Fig. 4A (averaged dB power changes from baseline) for the difference in repetition suppression and enhancement between the second and third presentation of a stimulus for repetitions with no lag (first row) and lag (second row) in control participants (left) and schizophrenia patients (right). There was no significant difference in repetition suppression or enhancement from the second to the third presentation of a stimulus within or between groups. (B) Line graphs show averaged power for gamma (top) and beta (bottom) frequencies over time per group for the first, second and third presentation of a stimulus in the NOLAG (first row) and LAG (second row) condition. HC = control participants, SCZ = schizophrenia patients.

Supplementary Figure 2. Correlational analyses. Scatterplots demonstrating correlations between AGE (left panel figures) and SEX (right panel figures; f = female, m = male) with (A) gamma-band suppression (43–98 Hz), (B) beta-band (12–33 Hz) enhancement, (C) C1m suppression in occipital ROIs as depicted in Fig. 4A, and (D) C1m suppression in thalamic ROIs as depicted in Fig. 4B. Data points include all 28 subjects.
